# Methodological Deficits in Diagnostic Research Using ‘-Omics’ Technologies: Evaluation of the QUADOMICS Tool and Quality of Recently Published Studies

**DOI:** 10.1371/journal.pone.0011419

**Published:** 2010-07-02

**Authors:** Lucy A. Parker, Noemí GómezSaez, Blanca Lumbreras, Miquel Porta, Ildefonso Hernández-Aguado

**Affiliations:** 1 Departamento de Salud Pública, Universidad Miguel Hernández, Alicante, Spain; 2 Centro de Investigación Biomédica en Red de Epidemiología y Salud Pública, (CIBERESP), Barcelona, Spain; 3 Institut Municipal d'Investigació Mèdica, Facultat de Medicina, Universitat Autònoma de Barcelona, Barcelona, Spain; HelmholtzZentrum München, Germany

## Abstract

**Background:**

QUADOMICS is an adaptation of QUADAS (a quality assessment tool for use in systematic reviews of diagnostic accuracy studies), which takes into account the particular challenges presented by ‘-omics’ based technologies. Our primary objective was to evaluate the applicability and consistency of QUADOMICS. Subsequently we evaluated and describe the methodological quality of a sample of recently published studies using the tool.

**Methodology/Principal Findings:**

45‘-omics’- based diagnostic studies were identified by systematic search of Pubmed using suitable MeSH terms (“Genomics”, “Sensitivity and specificity”, “Diagnosis”). Three investigators independently assessed the quality of the articles using QUADOMICS and met to compare observations and generate a consensus. Consistency and applicability was assessed by comparing each reviewer's original rating with the consensus. Methodological quality was described using the consensus rating. Agreement was above 80% for all three reviewers. Four items presented difficulties with application, mostly due to the lack of a clearly defined gold standard. Methodological quality of our sample was poor; studies met roughly half of the applied criteria (mean ± sd, 54.7±18.4%). Few studies were carried out in a population that mirrored the clinical situation in which the test would be used in practice, (6, 13.3%); none described patient recruitment sufficiently; and less than half described clinical and physiological factors that might influence the biomarker profile (20, 44.4%).

**Conclusions:**

The QUADOMICS tool can consistently be applied to diagnostic ‘-omics’ studies presently published in biomedical journals. A substantial proportion of reports in this research field fail to address design issues that are fundamental to make inferences relevant for patient care.

## Introduction

Technological advances in the past 20 years have permitted large-scale parallel measurements of biochemical and cellular constituents for study as a unified whole, spurring what may be referred to as the ‘-omics’ revolution. [1]–[3] By adding the suffix ‘-omics’, we can refer to the comprehensive study of almost any cellular constituent. For instance, transcriptomics refers to analysis of total mRNA expression and proteomics refers to the analysis of the proteome, the total protein content. The coupling of these high throughput technologies with computer-assisted discrimination systems may substantially influence the future of clinical diagnosis, leading to diagnostic tests based on multi-marker patterns, biomarker profiles or signatures, rather than on a single alteration [1], [4].

Despite rigorous and vigorous promotion of ‘-omics’ based technologies for diagnosis of human diseases, few of the many tests proposed have been introduced into clinical practice with clearly documented clinical benefits. [5]–[7] Analysis and interpretation of the diagnostic capacity of ‘-omics’ based technologies has presented unique challenges, [8] and reproducing the initial claims of diagnostic accuracy in independent populations has often proved complex. [9], [10] The apparent -but in fact artifactual- power to discriminate between diagnostic groups using ‘-omics’ technologies may actually be due to methodological features of the studies; most notably, differences in the pre-analytical procedures, [11] in the clinical or pathophysiological characteristics of the patients who provided the biological samples, [12]–[14] or simply chance. [15], [16] Consequently, in ‘-omics’ studies investigators must consider the potential genetic variation between different individuals, or how certain physiological characteristics (disease pathophysiology, stress, menstruation) could influence the serum protein profile of study participants. When designing and analysing their experiments, investigators must also consider the relative lack of stability of some of the cellular constituents detected by ‘-omics’ techniques, such as RNA degradation and repetitive freezing cycles. Furthermore, the tendency to develop or ‘discover’ the biomarker patterns using the available data, [17] rather than having a predefined hypothesis as to which biomarkers are likely to be involved, make these studies susceptible to overfitting [15], [16] (i.e., the apparent discrimination is due to chance and results cannot be reproduced in other populations). Additionally, ‘-omics’ technologies may be subject to limitations common to all diagnostic research. For example, one common problem in study design is the tendency to collect two groups of patients for discrimination separately (in what can be considered a diagnostic case-control study), instead of prospectively recruiting a group of patients with clinical suspicion of the disease under question, and then using the ‘-omics’ technology to discriminate between patients who are finally diagnosed with the disease and those who are not. [18], [19]

Achievement of all legitimate clinical and commercial interests requires that the provision of ‘-omics’-based diagnostic services be evidence based. [20] Tools for evaluating the quality of diagnostic research reports included in a systematic review, such as QUADAS, [21] have made a considerable impact in promoting evidence based diagnosis. Nevertheless, there is some concern that quality appraisal tools generic to all diagnostic tests may not be sufficiently adequate for this complex field, as such tools do not address the issues specific to the ‘-omics’ field previously mentioned. Consequently, we proposed an adaptation to the QUADAS guideline to take into account the particular challenges presented by ‘-omics’ based technologies. QUADOMICS [22] incorporates four new items addressing the type of sample used, differences in pre-analytical conditions, the clinical and physiological characteristics of the patients providing biological samples, and overfitting. Furthermore, it calls for users to classify each study into one of four phases of biomarker validation, according to the population in which the study is carried out. [23]–[25] In the first three phases a case control design may be used, and the objective could be to show discrimination between patients with overt disease and healthy individuals, to challenge the test with competing diagnoses, diverse co-morbidities or varying levels of disease severity, or to evaluate changes in diagnostic accuracy according to particular patient characteristics. However, in the fourth phase of evaluation, the test should be evaluated in a prospective series of individuals that reflect, with the maximum degree of fidelity, the clinical or public health setting where the test would be used.The evaluation of study phase was incorporated into QUADOMICS to increase recognition of issues related to the spectrum of patients studied, [26] and the requirements for synthesising results from studies in different phases when performing a meta-analysis. [27], [28]

As with any quality appraisal tool, it is essential that QUADOMICS be easy to apply and consistent, i.e., that independent users make analogous observations and judgements when appraising the same study. Accordingly, the primary objective of this study was to evaluate the applicability and consistency of the QUADOMICS tool by applying it to a broad selection of studies in triplicate. An associated secondary objective was the assessment of the methodological quality of the selection of recently published ‘-omics’ diagnostic studies, using this tool.

## Methods

The study consisted of two parts: 1) the evaluation of the applicability and consistency of the QUADOMICS tool, and 2) the evaluation of the methodological quality of a selection of recent published studies. The same selection of studies was used for both parts.

### Search Strategy

We identified original research articles by a systematic search of the Pubmed database combining the medical subject headings (MeSH) “Genomics”, “Sensitivity and specificity” and “Diagnosis”. The search was limited to articles published from 1^st^ January 2006 through June 17 2009 (the date of the search). The titles and abstracts of all potential articles were reviewed and articles were selected based on the following criteria: original research articles in which the key objective was to evaluate the diagnostic accuracy of an ‘-omics’ based test for use in clinical practice or a screening programme (we used the definition of ‘-omics’ applied in the development of QUADOMICS). [22] Studies which used ‘-omics’ techniques for the discovery of a biomarker pattern but then used standard laboratory techniques such as immunohistochemistry, ELISA or PCR to identify the biomarkers and validate the pattern were not selected. Furthermore, we only selected studies which presented a diagnostic accuracy measurement (e.g., sensitivity and specificity, area under ROC curve, diagnostic odds ratio, likelihood ratios) or that provided enough information for their calculation. Studies in which the main aim was to validate biomarkers for prognostic use or to predict the response to treatment were also excluded, as were articles published in languages other than English.

### Evaluation of the applicability and consistency of the QUADOMICS tool

Three investigators (LP, NG, BL) independently assessed the quality of all selected articles using the QUADOMICS tool. For reference, each reviewer was provided with a copy of the QUADOMICS publication, [22] the development of QUADAS publication [21] and the article evaluating QUADAS and providing some modifications to the items. [29] All three researchers met to compare their observations and generate the consensus rating after 8 articles had been reviewed, after 21, and finally after all 45; any disagreements were solved by discussion. During this process the authors explored the potential motives for the lack of agreement and discussed methods to improve the description of the item in the QUADOMICS guideline in order to avoid future discrepancies.

To evaluate the consistency of the QUADOMICS tool, we calculated the percentage agreement between each reviewer's original assessment and the consensus rating, both overall and for each item separately. We chose not to report Cohen's kappa statistic for inter-rater agreement because it is strongly influenced by the prevalence of each rating and can be misleading. [30] We regarded the consistency as “low” if agreement with the consensus was less than 60% for at least one reviewer, or if two or more reviewers had less than 80% agreement with the consensus. The reasons for limited consistency were evaluated and the item was reworked if necessary.

### Evaluation of the methodological quality of the selected articles

We used the consensus variables created during the evaluation of applicability and consistency of QUADOMICS to describe the methodological quality of the articles. As not all of the items were applied to every article (for instance, some criteria are only applied to articles in phase 4), we summarised the overall quality of each article by calculating the percentage of applied articles which scored positively. Finally, to identify if certain methodological short-comings were more common than others, we calculated the proportion of articles which met or failed to meet each item separately.

### Data analysis

Univariate descriptive statistics and 95% confidence intervals were computed as customary. [31], [32] All computations were carried out using STATA/SE 8.0 (StataCorp, College Station, TX, USA).

## Results and Discussion

The search strategy provided 164 potential articles, of which 59 were selected for full text revision and 45 were finally selected (Figure S1). The references of the 45 selected articles can be found in Annex S1 and a list of the study phase, study size, index test and reference standard of each study is found in Table S1.

### Applicability and consistency of QUADOMICS

Overall, the percentage agreement with the consensus rating was above 80% for all three reviewers (table 1). Of the 17 quality items, up to 4 were not applied to some of the articles. These included items 2 and 14, which should only be applied to studies in phase IV, as directed in the QUADOMICS background document. [22] Additionally, items 9 and 13 were only applied to some articles due to one or both of the following reasons: 1) the index test was almost exclusively performed after the reference diagnosis, and 2) many studies did not have an independent reference standard but, rather, the index test was tested against the diagnosis itself (which was also the criteria used by the authors to select the patients). For example, some studies selected a group of patients with the disease in question and a group of controls, either healthy individuals or with an alternative diagnosis. The lack of an independent reference test is a common problem in studies that seek to validate the diagnostic application of new ‘-omics’ based technologies and it contributed to difficulties in the application of the QUADOMICS items that refer to the reference standard. When possible, we applied these quality items by considering how and when the initial diagnosis was made, or how the diagnosis was ruled out in the controls. We decided that it would be unfair to score studies negatively for all items that mentioned the reference standard as they will not always be subject to the biases addressed by every quality item.

**Table 1:** Consistency in the application of the QUADOMICS tool to 45 diagnostic ‘-omics’ studies: % agreement with the consensus^1^.

	Reviewer 1		Reviewer 2		Reviewer 3	
	%	(95%CI)	%	(95%CI)	%	(95%CI)
Study Phase	91.1	(78.8–97.5)	97.8	(88.2–99.9)	73.3	(62.9–88.8)
1. Were selection criteria clearly described?	100		100		100	
2. Was the spectrum of patients representative of patients who will receive the test in practice?	95.2	(84.2–99.4)	100		97.7	(87.7–99.9)
3. Was the type of sample fully described?	86.7	(73.2–94.5)	91.1	(78.8–97.5)	77.8	(87.7–99.9)
4. Were the procedures and timing of biological sample collection with respect to clinical factors described with enough detail?						
4.1. Clinical and physiological factors	86.7	(73.2–94.5)	68.9	(53.2–81.4)	73.3	(58.1–85.4)
4.2. Diagnostic and treatment procedures.	88.9	(75.2–95.8)	86.7	(73.2–94.5)	80.0	(65.4–90.4)
5. Were handling and pre-analytical procedures reported in sufficient detail and similar for the whole sample? and, if differences in procedures were reported, was their effect on the results assessed?	64.4	(48.8–78.1)	93.3	(81.7–98.6)	88.9	(75.2–95.8)
6. Is the time period between the reference standard and the index test short enough to reasonably guarantee that the target condition did not change between the two tests?	68.9	(53.2–81.4)	84.4	(70.5–93.5)	53.3	(37.9–68.3)
7. Is the reference standard likely to correctly classify the target condition?	80.0	(65.4–90.4)	88.9	(75.2–95.8)	64.4	(48.8–78.3)
8. Did the whole sample or a random selection of the sample receive verification using a reference standard of diagnosis?	80.0	(65.4–90.4)	93.3	(81.7–98.6)	73.3	(58.1–85.4)
9. Did patients receive the same reference standard regardless of the result of the index test?	80.0	(65.4–90.4)	82.2	(67.9–92.0)	97.8	(88.2–99.9)
10. Was the execution of the index test described in sufficient detail to permit replication of the test?	84.4	(70.5–93.5)	77.8	(87.7–99.9)	88.9	(75.2–95.8)
11. Was the execution of the reference standard described in sufficient detail to permit its replication?	77.8	(87.7–99.9)	80.0	(65.4–90.4)	62.2	(46.5–76.2)
12. Were the index test results interpreted without knowledge of the results of the reference standard?	88.9	(75.2–95.8)	91.1	(78.8–97.5)	91.1	(78.8–97.5)
13. Were the reference standard results interpreted without knowledge of the results of the index test?	88.9	(75.2–95.8)	97.8	(88.2–99.9)	100	
14. Were the same clinical data available when test results were interpreted as would be available when the test is used in practice?	97.6	(87.4–99.9)	100		100	
15. Were uninterpretable/intermediate test results reported?	57.8	(42.2–72.0)	93.3	(81.7–98.6)	73.3	(58.1–85.4)
16. Is it likely that the presence of overfitting was avoided?	73.3	(58.1–85.4)	93.3	(81.7–98.6)	84.4	(70.5–93.5)
**Overall**	**83.0**	**(80.2–85.5)**	**89.9**	**(87.5–91.9)**	**82.3**	**(79.5–84.9)**

1 A consensus rating was achieved by discussion between the three reviewers for every item of each study separately.

When each item was analysed individually, four items -4.1, 6, 11 and 15- showed a low consistency according to our definition (one reviewer with less than 60% agreement with consensus, or 2+ reviewers with less than 80%). The motives for limited agreement are next discussed individually for each item.

#### Item 4.1: Were the procedures and timing of biological sample collection with respect to clinical factors described with enough detail? -Clinical and pathophysiological factors

There was some disagreement as to what constituted ‘enough detail’. Reporting sex and age of the patients in a descriptive table should not be considered sufficient to score positively. Ideally, authors should perform an analysis of the influence of procedures and timing of biological sample collection on the results of the test (example excerpt below). Nevertheless, in this review it was decided that studies scored positively as long as they provided some additional clinical information (apart from sex and age), such as cancer stage. It is advised that, before carrying out a systematic review, the authors discuss what is considered to be ‘enough detail’.

Example. Score positively:

*“… was employed to determine whether potentially confounding clinical variables such as patient age, sex, time from transplantation, HCV status, immunosuppressive therapy (…), and peripheral blood monocyte, lymphocyte, and neutrophil counts could be influencing gene-expression levels.”* [No. 17 in Annex S1]

#### Item 6: Is the time period between the reference standard and the index test short enough to reasonably guarantee that the target condition did not change between the two tests?

As discussed, most studies in ‘-omics’ technologies selected patients with established diagnosis and a control group, and used this classification as the reference standard. Thus, to evaluate disease progression bias [33] one should consider the time period between the initial diagnosis of the established condition and performance of the index test. This item is especially relevant for proteomics-based tests when the biomarker profile may be considerably different at different stages in disease. To score positively the diagnosis should be confirmed at the time of sample collection, and the disease stage should be noted or the time since diagnosis should be stated, so that disease progression bias can be evaluated (example excerpts below). If the authors fail to mention time since diagnosis this item should be marked unclear. If the authors mention time since diagnosis but the reviewer considers it to be too long (refer to QUADAS), [21] this item should be scored as no. If the test is based on a DNA microarray it is unlikely to be affected by the time since diagnosis and so this item will be scored as yes.

Example. Score positively:

*“At the time the sample was taken, all patients were classified by the clinician, according to standard criteria, as having active or inactive renal or systemic lupus.”* [No. 22 in Annex S1] *or “The clinical stage distribution of the 132 patients was as follows: stage I (n = 16); stage II (n = 56); stage III (n = 44); and stage IV (n = 16).”* [No. 43 in Annex S1]

Example. Score unclear:

*“Sera from pathologically confirmed lung cancer and benign tobacco-induced or tobacco-associated chronic lung disease patients were collected…”* [No. 12 in Annex S1]

#### Item 11: Was the execution of the reference standard described in sufficient detail to permit its replication?

The application of this item was made more complicated by the absence of an independent reference test in many of the studies. We evaluated whether the diagnostic criteria which gave rise to patient selection were described in enough detail. On several occasions, the diagnostic process for the cases with the disease of interest was described in sufficient detail; yet, there was relatively little information relating to how the authors established the absence of disease in the comparison group. Consistency was limited for this item because the reviewers dealt with this situation differently. We recommend that before carrying out a review, the authors discuss firstly whether they want to include studies that use prior diagnoses as the reference diagnosis, and secondly, if they choose to include them, what information should be given as a minimum to rule out the disease in the comparison group.

#### Item 15: Were uninterpretable/intermediate test results reported?

We experienced difficulties in evaluating this item as few studies mentioned uninterpretable results. We sought to apply the modification to this item made in the evaluation of QUADAS. *“If the authors do not report any uninterpretable/indeterminate/intermediate results, and if results are reported for all patients who were described as having been entered into the study then this item should also be scored as “yes”*. [21] Nevertheless, problems arose because it was difficult to judge if all patients described as having entered into the study contributed to the results presented, as often authors reported the diagnostic accuracy for different biomarker patterns (e.g., different protein peaks), without actually providing the crude patient numbers (example excerpt below). It was agreed that in this case we would mark the item “unclear”.

Example. Score positively:

*“…the test group had 52 patients and 33 controls.” → “Analyses of the spectra from the 85 testing samples showed that the classification algorithm correctly predicted 94% (80 of 85) of all of the samples, with 94% (49 of 52) of DLBCL samples and 94% (31 of 33) of the control samples. The specificity was 94% and the sensitivity was 94%.”* [No. 43 in Annex S1]

Example. Score unclear:

*“Cancers (62 samples) and controls (31 samples) were collected into identical tubes and processed in an identical manner.” → “Varying numbers of the most significant peaks were then used to develop ANNs to discriminate between cancer and non-cancer with 10-fold cross-validation. The ANNs developed using the seven most significant peaks performed best giving a sensitivity of 94% and specificity of 96%.”* [No. 37 in Annex S1]

### Quality of selected articles

Out of 45 included articles, 35 were considered to be in phase 1 (78%). Only 6 articles (13.3%) reflected the clinical situation in which the test would be used in practice, phase 4. This finding has important implications given that the case-control design used in phases I-III can lead to an overestimation of diagnostic accuracy. [34], [35]

There were 15 (33.3%) studies published in 2008, 13 (28.9%) each in 2006 and 2007, and 4 (8.9%) in 2009.

It is worth mentioning that the main goal in developing QUADOMICS, like QUADAS, was not for assessing the absolute quality in a cross-sectional sample of studies examining different technologies at different stages in development but, rather, for use in systematic reviews to identify differences in design and conduct that could lead to bias or variation in accuracy within a set of studies examining the same index test. Nevertheless, we have outlined how QUADOMICS can be tailored to suit the different phases of development and in such, any methodological shortcoming highlighted in our analysis was relevant considering the stage of development. Accordingly, up to four items were not applied to some of the selected articles and we evaluated the absolute quality of the studies by calculating the proportion of applied criteria that scored positively.

There was substantial variation in the number of quality criteria met by the selected articles, with one article meeting only 2 of 13 applied criteria (15.4%), [36] and another meeting 12 of 13 applied criteria (92.3%). [37]On average, the selected studies scored positively in just over half of the applied criteria (mean ± standard deviation, 54.7±18.4%). We have reported the percentage of applied criteria which scored positively to summarise the quality of the studies only. We do not believe that a critical threshold should be used when judging study quality [38]. We provide QUADOMICS as a tool that allows systematic reviewers and other readers to identify potential methodological weaknesses in a study, which could have biased the diagnostic accuracy, and therefore judge themselves whether study results are valid. The use of a critical threshold would not appropriately distinguish between a study with a single methodological shortcoming that completely invalidates the results, and a study that does not properly address a number of less influential items.

That being said, the methodological quality of the articles was generally poor, with numerous studies failing to address critical details. This in itself is a relevant finding because high quality studies are imperative if we are to ensure that the application of ‘-omics’ based diagnostic tests to clinical practice is evidence based. To identify the most common methodological short-comings, we explored the proportion of articles that met or failed to meet each item separately (Table 2). The most relevant findings are discussed in more detail below.

**Table 2:** Evaluation of the methodological quality of 45 diagnostic ‘-omics’ studies using the QUADOMICS tool.

Item	Yes	(%)	No	(%)	Unclear	(%)	N/A	(%)
1. Were selection criteria clearly described?	0	—	45	(100)	0	—	0	—
2. Was the spectrum of patients representative of patients who will receive the test in practice?	4	(8.9)	1	(2.2)	1	(2.2)	39	(86.7)
3. Was the type of sample fully described?	40	(88.9)	4	(8.9)	1	(2.2)	0	—
4. Were the procedures and timing of biological sample collection with respect to clinical factors described with enough detail?								
4.1. Clinical and physiological factors	20	(44.4)	25	(55.6)	0	—	0	—
4.2. Diagnostic and treatment procedures.	22	(48.9)	22	(48.9)	1	(2.2)	0	—
5. Were handling and pre-analytical procedures reported in sufficient detail and similar for the whole sample? and, if differences in procedures were reported, was their effect on the results assessed?	38	(84.4)	7	(15.6)	0	—	0	—
6. Is the time period between the reference standard and the index test short enough to reasonably guarantee that the target condition did not change between the two tests?	20	(44.4)	1	(2.2)	24	(53.3)	0	—
7. Is the reference standard likely to correctly classify the target condition?	33	(73.3)	6	(13.3)	6	(13.3)	0	—
8. Did the whole sample or a random selection of the sample receive verification using a reference standard of diagnosis?	24	(53.3)	14	(31.1)	7	(15.6)	0	(0.0)
9. Did patients receive the same reference standard regardless of the result of the index test?	1	(2.2)	0	—	0	—	44	(97.8)
11. Was the execution of the reference standard described in sufficient detail to permit its replication?	21	(46.7)	24	(53.3)	0	—	0	—
12. Were the index test results interpreted without knowledge of the results of the reference standard?	20	(44.4)	25	(55.6)	0	—	0	—
13. Were the reference standard results interpreted without knowledge of the results of the index test?	6	(13.3)	0	—	0	—	39	(86.7)
14. Were the same clinical data available when test results were interpreted as would be available when the test is used in practice?	5	(11.1)	1	(2.2)	0	—	39	(86.7)
15. Were uninterpretable/intermediate test results reported?	33	(73.3)	2	(4.4)	10	(22.2)	0	—
16. Is it likely that the presence of overfitting was avoided?	20	(44.4)	22	(48.9)	3	(6.7)	0	—

#### Aspects relating to the patient population and samples studied (Items 1–5)

In general, the description of the sample population was poor and none of the articles scored positively for item 1 due to the absence of a flow diagram describing the flow of patients in the selection process. The limited description of the patient population observed in these studies was disconcerting as this information is essential in order to assess external validity. Interestingly, even one of the phase 4 studies, scored negatively for the item on patient spectrum (item 2, example excerpt below). This study sought to validate a proteomics based urine test for the diagnosis of ovarian cancer. [39] Although it was considered to be phase 4 due to the inclusion of a consecutive series of patients, it is likely that by selecting women undergoing surgery the study selected a more severely diseased patient population than would normally receive the urine based test:

Example:

*“Urine samples and paired blood samples were prospectively collected from 209 consecutive women admitted for an exploratory laparotomy for an ovarian neoplasm at the Gynaecological Department at Rigshospitalet, Copenhagen between June 2006 and August 2007.”* [No. 25 in Annex S1]

Only half of the studies considered the diagnostic or treatment procedures undergone by the patient before the sample was taken (Item 4.2: 22, 49.9%), and even fewer described the clinical and pathophysiological factors that might influence the biomarker profile [13], [14] (Item 4.1: 20 studies, 44.4%). Most articles clearly described the type of sample used and the pre-analytical procedures in sample preparation (Item 3: 40, 88.9%, Item 5: 38, 84.4%).

#### Aspects relating to the test being evaluated (Items 10, 13, 14)

19 (42.2%) studies did not describe the index test in enough detail (Item 10). Less than half of the studies (Item 13: 20, 44.4%) mentioned whether the index test result was interpreted without knowledge of the reference standard; such omission suggests that review bias was possible. [19], [26] On the other hand, one of the phase 4 studies was subject to a kind of over blinding, and scored negatively in item 14 (example excerpt below). This study evaluated a gene expression profile for the identification of the tissue of origin in the case of metastatic, poorly differentiated specimens. [40] Although blinding of the reference diagnosis is necessary to avoid review bias, in clinical practice the clinician interpreting the test would have access to details such as patient sex and tumour pathology.

Example. *“… investigators who interpreted the Pathwork Tissue of Origin Test results for making a tissue determination were blinded to patient sex, histology, or morphology information, and reference diagnosis”* [No. 21 in Annex S1]

#### Aspects relating to the reference test (Items 6, 11)

Over half of the articles did not describe the reference test in enough detail (Item 11: 21, 46.7%). As mentioned earlier many of the articles did not actually include an independent reference test. In this case we evaluated the diagnosis of the target condition or selection criteria for the comparison group. Furthermore, over half of the articles failed to mention any time period with regard to diagnosis, making it difficult to judge whether the target condition could have changed (item 6: 24, 53.3% unclear).

#### Overfitting (Item 16)

22 (48.9%) studies did not effectively control for overfitting, and in 3 studies (6.7%) it was not clear if validation was carried out in samples from the same patients in which the model was built. Only studies that validated their biomarker signature in an independent set of patient samples scored positively for this item; i.e., studies that performed internal validation using cross validation alone did not score positively. We deem this an important finding because it is likely that the results presented in these studies are overly optimistic [41] and may not be reproducible in other patient populations. [42]

Finally, there was no apparent change in the proportion of studies meeting each item separately over the 4 years studies (data not shown), but numbers were small.

### Conclusions

In this study we showed that three reviewers could apply the QUADOMICS tool to a broad sample of diagnostic ‘-omics’ studies with reasonable consistency. A small number of items were difficult to apply to studies that did not use an independent test for determining the reference diagnosis. This problem with item applicability arose in studies which used a healthy or alternative diagnosis comparison group and, thus, it was closely linked to the study phase of the articles (phases I–III). On one hand, the importance of this problem is limited because systematic reviews and meta-analyses carried out to inform decision makers of the evidence supporting the use of a test in clinical practice should focus on studies with more clinically relevant populations (phase IV). On the other hand, it is highly important that the quality of early phase studies is adequately assessed in order to weigh up the evidence and decide if it is a sensible use of resources to proceed to studies in more clinically relevant populations. Here, we have outlined how the QUADOMICS criteria can be applied to these studies.

In practice the QUADOMICS guideline will be used to evaluate studies included in a systematic review and, therefore, studies should all be addressing the same diagnostic question, and be in the same phase. Similar to QUADAS, [21] reviewers should tailor the guideline to suit their specific review question. For example, if they want to assess the utility of the test for use in clinical practice, they should only include phase IV studies, and make some decisions before evaluating the studies (e.g., what should be the appropriate reference standard, how much information is considered to be ‘sufficient detail’ or how long is too long for the time period between reference and index test). On the other hand, a review carried out to assess the preliminary evidence in favour of a new ‘-omics’ test in order to judge whether it would be sensible or appropriate to carry out a large scale prospective evaluation may include studies from earlier phases which use the case-control type design. While it would extremely important to consider differences between the two diagnostic groups with regard to pre-analytical conditions (item 5), or the clinical characteristics of the patients providing samples (item 4), it would be inappropriate to score a study negatively because it does not meet item 2 (‘Was the spectrum of patients representative of patients who will receive the test in practice?’). In this case the tailoring of the guideline would involve eliminating the items that are not applicable as well as making decisions as how specific items should be scored. By applying QUADOMICS to a broad range of articles from different subjects, we have shown that it is flexible, and we believe that the ability to be tailored to the different study phases is one of its key strengths.

The methodological quality of our selection of 45 ‘-omics’ based diagnostic studies was poor. It is alarming, for example, that none of the studies included a flow diagram describing the patient recruitment process; such diagrams are also strongly recommended in the Standards for Reporting of Diagnostic Accuracy (STARD) publication. [43] This deficiency is not specific to the ‘-omics’ field; for instance, a recent review of commercial tests for HIV, TB or malaria showed that only 13% of studies reviewed met the STARD criterion which recommends the flow diagram. [44] This issue is in fact a reporting item and therefore only indirectly linked to quality. Studies that meet this criterion do not automatically have clinically relevant populations, yet in studies that do not clearly describe patient recruitment it is impossible to evaluate whether the results are applicable to our context. It is arguable that reporting items have no place in instruments measuring methodological quality however, despite increased sensitisation to issues related to the quality of reporting, diagnostic research remains poorly reported [45] and evaluating methodological quality relies on transparent and good quality reporting. In such we feel that such items do help draw attention of the readers to potential methodological limitations, and thus reduce assumptions that the methodology was sound.

There were other threats to the validity of the studies. For instance, it is now recognised that patient treatment regimes or other clinical and pathophysiological characteristics may influence the parameters studied, such as proteins, and thus bias ‘-omics’ studies. [13], [14], [46], [47] Nevertheless, few of the studies we assessed actually reported these details, let alone analysed their potential effect. Furthermore, in nearly half of the articles the diagnostic model was not validated in an independent set of patients; such shortfall may lead to overfitting and the production of results that are not reproducible. Coupled with the fact that very few of the studies were actually carried out in a consecutive set of patients with clinical suspicion of the disease in question, the problem illustrates the relative lack of attention paid in ‘-omics’ research to design issues that are fundamental when we aim at making inferences relevant for patient care.

One limitation of this study is the external validity of our assessment of the quality of recent articles published in this field, our secondary objective. We do not presume to have included all diagnostic ‘-omics’ studies published in 2006 through 2009. While our sample was not restricted to any particular field or technique, it is clear that it was limited to reports indexed by Medline, and adequately tagged with the selected MeSH terms. Nevertheless for our primary objective, we feel that the selected sample was sufficiently diverse to adequately assess the applicability and consistency of the QUADOMICS tool.

Another issue is related to the three reviewers used to evaluate the consistency and applicability of QUADOMICS. While the three reviewers had different backgrounds and varying levels of research experience, in principle it would have been beneficial to include a larger number of reviewers with a wider knowledge of the diseases of interest. Furthermore, two of the three observers were involved in the development of the tool, and hence may have found the tool easier to apply. However, in practice QUADOMICS will be used to evaluate the quality of studies addressing the same diagnostic question and reviewers will decide a priori how each item should be scored. In such situations it is likely that application would be more straightforward and that reviewer observations would be more consistent. Here we provide an evaluation of the tool in general, rather than for every subject separately, because at this stage in the development of QUADOMICS, we felt it was important to ensure the tool was applicable to a broad range of real studies.

For ethical, clinical and economic reasons, the application of ‘-omics’ based tests in clinical practice requires valid and reliable research that can be reproduced in clinically relevant patient populations. [23]–[25] While some of the methodological deficiencies we described were linked to the specific peculiarities of ‘-omics’ based research, other important aspects -which have long been considered fundamental in traditional diagnostic research, such as the description of the index test and test reproducibility- are being overlooked in ‘-omics’ research. The QUADOMICS tool was proposed for the assessment of the methodological quality of diagnostic research using ‘-omics’ based technology. [22] We show that the tool can consistently be applied to a broad range of these studies. Furthermore, we hope that it will help sensitize researchers, clinicians and other decision makers to the serious threats to the validity inherent to this type of research, and ensure that the provision of ‘-omics’ tests to the clinic is evidence based.

## Supporting Information

Figure S1 Flow diagram of search and selection process.
(0.26 MB TIF)

Annex S1 References of the 45 articles evaluated.
(0.04 MB DOC)

Table S1 Characteristics of 45 studies evaluating the diagnostic use of an ‘-omics’ based test.
(0.10 MB DOC)
