# Supplementary figures and images for: Methodological Deficits in Diagnostic Research Using ‘-Omics’ Technologies: Evaluation of the QUADOMICS Tool and Quality of Recently Published Studies
Source: PLoS One. 2010 Jul 2;5(7):e11419. doi: 10.1371/journal.pone.0011419 (PMC2896422; doi:10.1371/journal.pone.0011419)

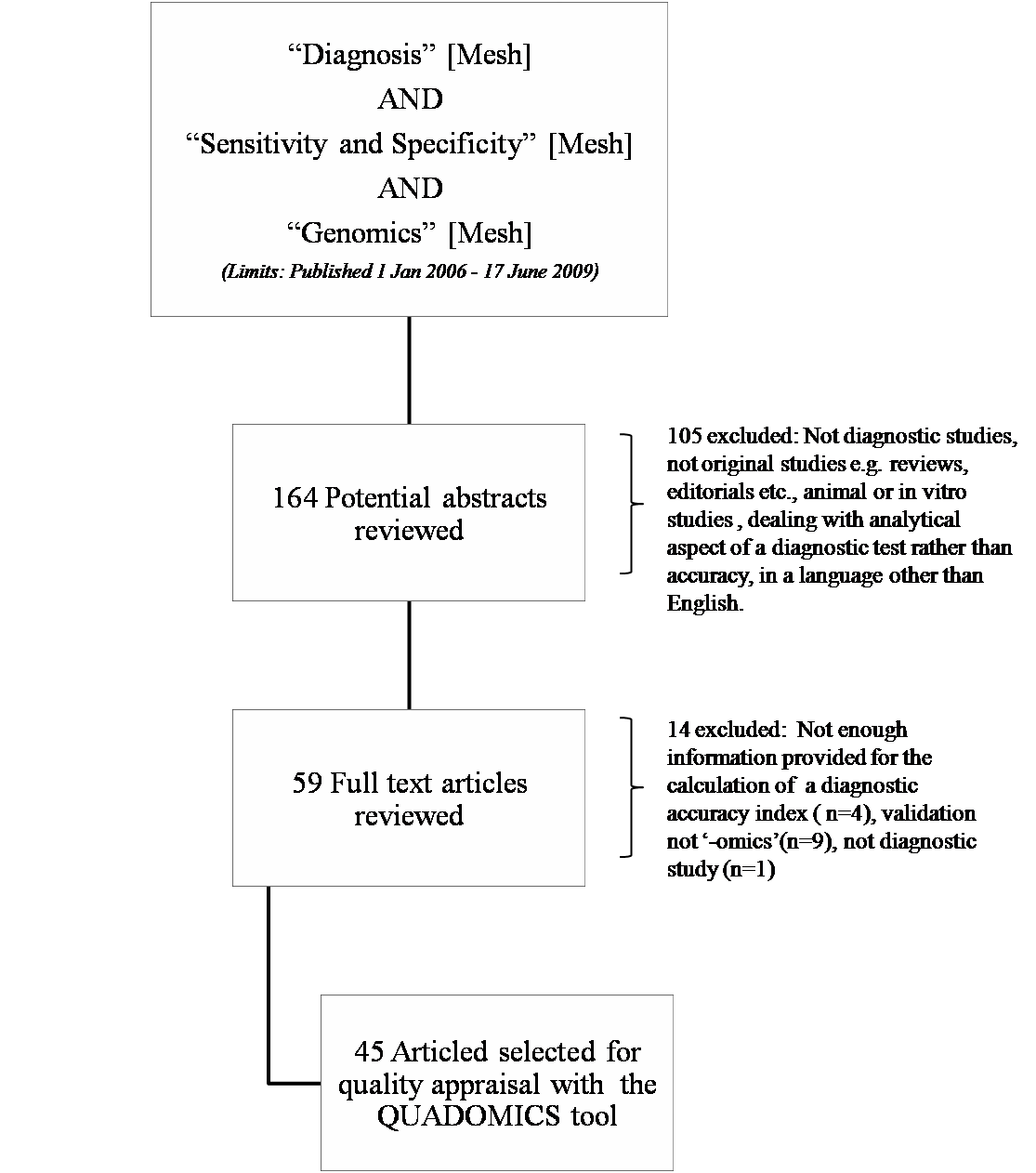

Supplement: Figure S1 — Flow diagram of search and selection process. (0.26 MB TIF) [file pone.0011419.s001.tif]
