# Supplementary material for: Methodological Deficits in Diagnostic Research Using ‘-Omics’ Technologies: Evaluation of the QUADOMICS Tool and Quality of Recently Published Studies
Source: PLoS One. 2010 Jul 2;5(7):e11419. doi: 10.1371/journal.pone.0011419 (PMC2896422; doi:10.1371/journal.pone.0011419)
Supplement: Annex S1 — References of the 45 articles evaluated. (0.04 MB DOC) [file pone.0011419.s002.doc]

**Annex 1: List of 45 articles evaluated.**

1. Belluco C, Petricoin EF, Mammano E, Facchiano F, Ross-Rucker S, Nitti D et al. Serum proteomic analysis identifies a highly sensitive and specific discriminatory pattern in stage 1 breast cancer. Ann Surg Oncol. 2007;14:2470-6
2. Bhattacharyya S, Epstein J, Suva LJ. Biomarkers that discriminate multiple myeloma patients with or without skeletal involvement detected using SELDI-TOF mass spectrometry and statistical and machine learning tools. Dis Markers. 2006;22:245-55
3. Bons JA, Drent M, Bouwman FG, Mariman EC, van Dieijen-Visser MP, Wodzig WK. Potential biomarkers for diagnosis of sarcoidosis using proteomics in serum. Respir Med. 2007;101:1687-95
4. Buhimschi CS, Bhandari V, Hamar BD, Bahtiyar MO, Zhao G, Sfakianaki AK et al. Proteomic profiling of the amniotic fluid to detect inflammation, infection, and neonatal sepsis. PLoS Med. 2007;4:e18
5. Buhimschi IA, Zambrano E, Pettker CM, Bahtiyar MO, Paidas M, Rosenberg VA et al. Using proteomic analysis of the human amniotic fluid to identify histologic chorioamnionitis. Obstet Gynecol. 2008 ;111:403-12
6. Cepek L, Brechlin P, Steinacker P, Mollenhauer B, Klingebiel E, Bibl M et al. Proteomic analysis of the cerebrospinal fluid of patients with Creutzfeldt-Jakob disease. Dement Geriatr Cogn Disord. 2007;23:22-8
7. Das S, Maeso PA, Becker AM, Prosser JD, Adam BL, Kountakis SE. Proteomics blood testing to distinguish chronic rhinosinusitis subtypes. Laryngoscope. 2008;118:2231-4
8. Finehout EJ, Franck Z, Choe LH, Relkin N, Lee KH. Cerebrospinal fluid proteomic biomarkers for Alzheimer's disease. Ann Neurol. 2007;61:120-9
9. Han KQ, Huang G, Gao CF, Wang XL, Ma B, Sun LQ et al. Identification of lung cancer patients by serum protein profiling using surface-enhanced laser desorption/ionization time-of-flight mass spectrometry. Am J Clin Oncol. 2008;31:133-9
10. He QY, Zhu R, Lei T, Ng MY, Luk JM, Sham P et al. Toward the proteomic identification of biomarkers for the prediction of HBV related hepatocellular carcinoma. J Cell Biochem. 2008;103:740-52
11. Hong M, Zhang X, Hu Y, Wang H, He W, Mei H et al. The potential biomarkers for thromboembolism detected by SELDI-TOF-MS. Thromb Res. 2009;123:556-64
12. Jacot W, Lhermitte L, Dossat N, Pujol JL, Molinari N, Daurès JP et al. Serum proteomic profiling of lung cancer in high-risk groups and determination of clinical outcomes. J Thorac Oncol. 2008;3:840-50.
13. Kyselova Z, Mechref Y, Kang P, Goetz JA, Dobrolecki LE, Sledge GW et al. Breast cancer diagnosis and prognosis through quantitative measurements of serum glycan profiles. Clin Chem. 2008;54:1166-75
14. Leiserowitz GS, Lebrilla C, Miyamoto S, An HJ, Duong H, Kirmiz C et al. Glycomics analysis of serum: a potential new biomarker for ovarian cancer? Int J Gynecol Cancer. 2008 ;18:470-5
15. Liang Y, Fang M, Li J, Liu CB, Rudd JA, Kung HF et al. Serum proteomic patterns for gastric lesions as revealed by SELDI mass spectrometry. Exp Mol Pathol. 2006;81:176-80
16. Lin YW, Lai HC, Lin CY, Chiou Jy, Shui HA, Chang CC et al. Plasma proteomic profiling for detecting and differentiating in situ and invasive carcinomas of the uterine cervix. Int J Gynecol Cancer. 2006; 16:1216-24
17. Martínez-Llordella M, Lozano JJ, Puig-Pey I, Orlando G, Tisone G, Lerut J et al. Using transcriptional profiling to develop a diagnostic test of operational tolerance in liver transplant recipients. J Clin Invest. 2008;118:2845-57
18. McLerran D, Grizzle WE, Feng Z, Bigbee WL, Banez LL, Cazares LH et al. Analytical validation of serum proteomic profiling for diagnosis of prostate cancer: sources of sample bias. Clin Chem. 2008;54:44-52
19. McLerran D, Grizzle WE, Feng Z, Thompson IM, Bigbee WL, Cazares LH et al. SELDI-TOF MS whole serum proteomic profiling with IMAC surface does not reliably detect prostate cancer. Clin Chem. 2008;54:53-60
20. Meuwis MA, Fillet M, Geurts P, de Seny D, Lutteri L, Chapelle JP et al. Biomarker discovery for inflammatory bowel disease, using proteomic serum profiling. Biochem Pharmacol. 2007;73:1422-33
21. Monzon FA, Lyons-Weiler M, Buturovic LJ, Rigl CT, Henner WD, Sciulli C et al. Multicenter validation of a 1,550-gene expression profile for identification of tumor tissue of origin. J Clin Oncol. 2009;27:2503-8
22. Mosley K, Tam FWK, Edwards RJ, Crozier J, Pusey CD, Lightstone L. Urinary proteomic profiles distinguish between active and inactive lupus nephritis. Rheumatology. 2006;45:1497-504
23. Ordway JM, Budiman MA, Korshunova Y, Maloney RK, Bedell JA, Citek RW et al. Identification of novel high-frequency DNA methylation changes in breast cancer. PLoS One. 2007;2:e1314
24. Pasinetti GM, Unger LH, Lange DJ, Yemul S, Deng H, Yuan X et al. Identification of potential CSF biomarkers in ALS. Neurology. 2006;66:1218-22
25. Petri AL, Simonsen AH, Yip TT, Hogdall E, Fung ET, Lundvall L et al. Three new potential ovarian cancer biomarkers detected in human urine with equalizer bead technology. Acta Obstet Gynecol Scand. 2009;88:18-26
26. Poon TCW, Sung JJY, Chow SM, Ng EKW, Yu ACW, Chu ESH et al. Diagnosis of Gastric cancer by serum proteomic fingerprinting. Gastroenterology. 2006;130:1858-64.
27. Reddy A, Wang H, Yu H, Bonates TO, Gulabani V, Azok J et al. Logical Analysis of Data (LAD) model for the early diagnosis of acute ischemic stroke. BMC Med Inform Decis Mak. 2008;8:30
28. Ren H, Du N, Liu G, Hu HT, Tian W, Deng ZP et al. Analysis of variabilities of serum proteomic spectra in patients with gastric cancer before and after operation. World J Gastroenterol. 2006;12:2789-92
29. Sanders ME, Dias EC, Xu BJ, Mobley JA, Billheimer D, Roder H et al. Differentiating proteomic biomarkers in breast cancer by laser capture microdissection and MALDI MS. J Proteome Res. 2008;7:1500-7
30. Scarlett CJ, Smith RC, Saxby A, Nielson A, Samra JS, Wilson SR et al Proteomic Classification of Pancreatic Adenocarcinoma Tissue Using Protein Chip Technology. Gastroenterology. 2006;130:1670–8
31. Sogawa K, Itoga S, Tomonaga T, Nomura F. Diagnostic values of surface-enhanced laser desorption/ionization technology for screening of habitual drinkers. Alcohol Clin Exp Res. 2007;31:S22-6.
32. Srinivasan R, Daniels J, Fusaro V, Lundqvist A, Killian JK, Geho D et al. Accurate diagnosis of acute graft versus host disease using serum proteomic pattern analysis. Exp Hematol. 2006;34:796-801.
33. Su Y, Shen J, Qian H, Ma H, Ji J, Ma H et al. Diagnosis of gastric cancer using decision tree classification of mass spectral data. Cancer Sci. 2007;98:37-43
34. Theodorescu D, Wittke S, Ross MM, Walden M, Conaway M, Just I et al. Discovery and validation of new protein biomarkers for urothelial cancer: a prospective analysis. Lancet Oncol. 2006;7:230-40.
35. Wada-Isoe K, Michio K, Imamura K, Nakaso K, Kusumi M, Kowa H et al. Serum proteomic profiling of dementia with Lewy bodies: diagnostic potential of SELDI-TOF MS analysis. J Neural Transm. 2007;114:1579-83
36. Wang L, Zheng W, Mu L, Zhang SZ. Identifying biomarkers of endometriosis using serum protein fingerprinting and artificial neural networks. Int J Gynaecol Obstet. 2008;101:253-8
37. Ward DG, Suggett N, Cheng Y, Wei W, Johnson H, Billingham LJ et al. Identification of serum biomarkers for colon cancer by proteomic analysis. Br J Cancer. 2006;94:1898-905
38. Wei YS, Zheng YH, Liang WB, Zhang JZ, Yang ZH, Lv ML et al. Identification of serum biomarkers for nasopharyngeal carcinoma by proteomic analysis. Cancer. 2008;112:544-51.
39. Weissinger EM, Schiffer E, Hertenstein B, Ferrara JL, Holler E, Stadler M et al. Proteomic patterns predict acute graft-versus-host disease after allogeneic hematopoietic stem cell transplantation. Blood. 2007;109:5511-9
40. Wu C, Wang Z, Liu L, Zhao P, Wang W, Yao D et al. Surface enhanced laser desorption/ionization profiling: New diagnostic method of HBV-related hepatocellular carcinoma. J Gastroenterol Hepatol. 2009;24:55-62
41. Wu SP, Lin YW, Lai HC, Chu TY, Kuo YL, Liu HS. SELDI-TOF MS profiling of plasma proteins in ovarian cancer. Taiwan J Obstet Gynecol. 2006;45:26-32
42. Yildiz PB, Shyr Y, Rahman JS, Wardwell NR, Zimmerman LJ, Shakhtour B et al. Diagnostic accuracy of MALDI mass spectrometric analysis of unfractionated serum in lung cancer. J Thorac Oncol. 2007;2:893-901
43. Zhang X, Wang B, Zhang XS, Li ZM, Guan ZZ, Jiang WQ. Serum diagnosis of diffuse large B-cell lymphomas and further identification of response to therapy using SELDI-TOF-MS and tree analysis patterning. BMC Cancer. 2007;7:235-46
44. Zhou L, Cheng L, Tao L, Jia X, Lu Y, Liao P.Detection of hypopharyngeal squamous cell carcinoma using serum proteomics. Acta Oto-laryngol. 2006;126:853-60
45. Zhu LR, Zhang WY, Yu L, Zheng YH, Hu J, Liao QP. Proteiomic patterns for endometrial cancer using SELDI-TOF-MS. J Zhejiang Univ Sci B. 2008;9:286-90
