# Supplementary material for: Methodological Deficits in Diagnostic Research Using ‘-Omics’ Technologies: Evaluation of the QUADOMICS Tool and Quality of Recently Published Studies
Source: PLoS One. 2010 Jul 2;5(7):e11419. doi: 10.1371/journal.pone.0011419 (PMC2896422; doi:10.1371/journal.pone.0011419)
Supplement: Table S1 — Characteristics of 45 studies evaluating the diagnostic use of an ‘-omics’ based test. (0.10 MB DOC) [file pone.0011419.s003.doc]

**Table S1**: Characteristics of 45 studies evaluating the diagnostic use of an ‘-omics’ based test.

| **1ST AUTHOR** | **YEAR** | **JOURNAL** | **PHASE** | **N** | **TARGET DISORDER** | **INDEX TEST** | **REFERENCE STANDARD** |
| --- | --- | --- | --- | --- | --- | --- | --- |
| Belluco | 2007 | Ann Surg Oncol | I | 310 | Breast cancer | Serum proteomic profiles using SELDI-TOF-MS | Pathologically proven disease and mammography negative controls |
| Bhattacharayya | 2006 | Dis Markers | I | 48 | Bone involvement in multiple myeloma | Serum proteomic profiles using SELDI-TOF-MS | Established diagnosis with radiological evidence of bone involvement |
| Bons | 2007 | Respir Med | I | 70 | Sarcoidosis | Serum proteomic profiles using SELDI-TOF-MS | Established diagnosis |
| Buhimschi | 2008 | Obstet Gynecol | IV | 158 | Chlorioamnionitis | Amniotic fluid proteomic fingerprint using SELDI-TOF-MS: Mass Restricted score | Histology |
| Buhimschi | 2007 | PLoS Med | IV | 169 | Neonatal sepsis | Amniotic fluid proteomic fingerprint using SELDI-TOF-MS: Mass Restricted score | Clinical symptoms and laboratory analysis |
| Cepek | 2007 | Dement Geriatr Cogn Disord | I | 28 | Creutzfeldt-jakob disease | CSF proteomic profile using 2DGE | Established diagnosis |
| Das | 2008 | Laryngoscope | IV | 42 | Chronic rhinosinusitis subtypes | Serum proteomic profiles using SELDI-TOF-MS | Fulfilment of established diagnostic criteria depending on subtype |
| Finehout | 2007 | Ann Neurol | I | 96 | Alzheimer's disease | CSF proteomic profile using 2DGE | Established diagnosis |
| Han | 2008 | Am J Clin Oncol | I | 253 | Lung cancer | Serum proteomic profiles using SELDI-TOF-MS | Established diagnosis and healthy controls |
| He | 2008 | J Cell Biochem. | I | 164 | HBV related hepatocellular carcinoma | Serum proteomic profiles using SELDI-TOF-MS | Established diagnoses and healthy controls |
| Hong | 2009 | Thromb Res | I | 69 | Thromboembolism | Plasma proteomic profiles usinf SELDI-TOF-MS | Established diagnosis and healthy controls |
| Jacot | 2008 | J Thorac Oncol | II | 170 | Lung cancer | Serum proteomic profiles using SELDI-TOF-MS | Established diagnoses |
| Kyselova | 2008 | Clin Chem | I | 109 | Breast cancer | Serum glycomic profile using MALDI-MS | Established diagnosis and healthy controls |
| Leiserowitz | 2008 | Int J Gynecol Cancer | I | 72 | Ovarian cancer | Serum glycomic profile using MALDI-FTMS | Established diagnosis and normal controls |
| Liang | 2006 | Exp Mol Pathol | I | 127 | Gastric lesions | Serum proteomic profiles using SELDI-MS | Established diagnosis and healthy controls |
| Lin | 2006 | Int J Gynecol Cancer | I | 129 | Cervical cancer | Plasma proteomic profiles usinf SELDI-TOF-MS | Established diagnosis and healthy controls |
| Martínez-Llordella | 2008 | J Clin Invest | I | 96 | Tolerance in liver transplants | Gene expression profiles in PBMC using oligonucleotide microarray | Established diagnosis |
| McLerran | 2008 | Clin Chem | II | 400 | Prostate cancer | Serum proteomic profiles using SELDI-TOF-MS | Established diagnoses |
| McLerran | 2008 | Clin Chem | I | 544 | Prostate cancer | Serum proteomic profiles using SELDI-TOF-MS | Established diagnoses and normal controls |
| Meuwis | 2007 | Biochem Pharmacol | II | 120 | Inflammatory bowel disease | Serum proteomic profiles using SELDI-TOF-MS | Established diagnosis |
| Monzon | 2009 | J Clin Oncol | IV | 547 | Tumour tissue of origin | Gene expression profiles in tissue using microarray | Histology |
| Mosley | 2006 | Rheumatology | I | 57 | Active lupus nephritis | Urinary proteomic profiles using SELDI-TOF-MS | Established diagnoses |
| Ordway | 2007 | PLoS One | I | 230 | Breast cancer | DNA methylation profiles is tissue using microarray | Established diagnosis and normal controls |
| Pasinetti | 2006 | Neurology | I | 102 | Amyotrophic Lateral Sclerosis | CSF proteomic profile using SELDI-MS | Established diagnoses and normal controls |
| Petri | 2009 | Acta Obstet Gynecol Scand | IV | 209 | Ovarian cancer | Urinary proteomic profiles using SELDI-TOF-MS | Surgery and histopathology |
| Poon | 2006 | Gastroenterology | I | 123 | Gastric cancer | Serum proteomic profiles using SELDI-MS | Established diagnosis and healthy controls |
| Reddy | 2008 | BMC Med Inform Decis Mak | I | 130 | Ischemic stroke | Logistic analysis of data applied to serum proteomic profiles | Established diagnosis and healthy samples from blood bank |
| Ren | 2006 | World J Gastroenterol | I | 86 | Gastric cancer | Serum proteomic profiles using SELDI-TOF-MS | Established diagnosis and healthy controls |
| Sanders | 2008 | J Proteome Res | I | 289 | Breast cancer | Tissue proteomic patterns using MALDI-MS | Established diagnosis and reduction mammoplasty specimens |
| Scarlett | 2006 | Gastroenterology | I | 50 | Pancreatic adenocarcinoma | Tissue proteomic patterns using SELDI-TOF-MS | Histology |
| Sogawa | 2007 | Alcohol Clin Exp Res | I | 75 | Alcoholism | Serum proteomic profiles using SELDI-TOF-MS | Questionnaire |
| Srinivasen | 2006 | Exp Hematol | I | 34 | Graft-versus-host disease | Serum proteomic profiles using SELDI-TOF-MS | Clinical findings and partial histopathological analysis |
| Su | 2007 | Cancer Sci | I | 245 | Gastric cancer | Serum proteomic profiles using SELDI-TOF-MS | Established diagnoses and healthy controls |
| Theodorescu | 2006 | Lancet Oncol | III | 655 | Urothelial cancer | Urinary proteomic profiles using CE-MS | Various established diagnoses and healthy controls |
| Wada-Isoe | 2007 | J Neural Transm | I | 52 | Dementia with Lewis bodies | Serum proteomic profiles using SELDI-TOF-MS | Established diagnosis |
| Wang | 2008 | Int J Gynaecol Obstet | I | 66 | Endometriosis | Serum proteomic profiles using SELDI-TOF-MS | Established diagnosis and healthy controls |
| Ward | 2006 | Br J Cancer | I | 93 | Colorectal cancer | Serum proteomic profiles using SELDI-MS | Established diagnosis and healthy controls |
| Wei | 2008 | Cancer | I | 168 | Naspharyngeal carcinoma | Serum proteomic profiles using SELDI-TOF-MS | Established diagnosis and healthy volunteers |
| Weissinger | 2007 | Blood | IV | 141 | Graft-versus-host disease | Urinary proteomic profiles using CE-MS | Histopathologic examination of tissue biopsies |
| Wu | 2006 | Taiwan J Obstet Gynecol | I | 65 | Ovarian cancer | Plasma proteomic profiles using SELDI-TOF-MS | Established diagnosis and healthy controls |
| Wu | 2009 | J Gastroenterol Hepatol | I | 59 | Hepatocellular carcinoma | Serum proteomic profiles using SELDI-TOF-MS | Established diagnoses |
| Yildiz | 2007 | J Thorac Oncol | I | 288 | Lung cancer | Serum proteomic profiles using MALDI-MS | Established diagnosis and healthy controls |
| Zhang | 2007 | BMC Cancer | I | 207 | Large B-cell lymphomas | Serum proteomic profiles using SELDI-TOF-MS | Established diagnosis and healthy controls |
| Zhou | 2006 | Acta Oto-laryngol | I | 100 | Hypopharngeal squamous cell carcinoma | Serum proteomic profiles using SELDI-TOF-MS | Established diagnosis and healthy controls |
| Zhu | 2008 | J Zhejiang Univ Sci B | I | 100 | Endometrial cancer | Serum proteomic profiles using SELDI-TOF-MS | Established diagnosis and healthy volunteers |

**Abbreviations:** 2DGE, two-dimensional gel electrophoresis; CE-MS, capillary electrophoresis coupled online to mass spectrometry; CSF, cerebrospinal fluid; HBV, hepatitis B virus; MALDI-MS, matrix-assisted laser desorption ionization mass spectrometry; MALDI-FTMS, matrix-assisted laser desorption ionization fourier transformation mass spectrometry; PBMC, Peripheral blood mononuclear cell; SELDI-TOF-MS, surface enhanced laser desorption ionization time of flight mass spectrometry.
